# Hair cortisol and dehydroepiandrosterone concentrations in naturally *Taenia solium* infected pigs in Tanzania

**DOI:** 10.1016/j.ygcen.2017.03.007

**Published:** 2017-05-15

**Authors:** Chiara Trevisan, Marta Montillo, Alberto Prandi, Ernatus M. Mkupasi, Helena A. Ngowi, Maria V. Johansen

**Affiliations:** a Department of Veterinary and Animal Sciences, University of Copenhagen, Dyrlægevej 100, 1870 Frederiksberg C, Denmark; b Department of Agricultural, Food, Environmental and Animal Sciences, University of Udine, Via Sondrio 2/A, 33100 Udine, Italy; c Department of Veterinary Medicine and Public Health, Sokoine University of Agriculture, P.O. Box 3021, Morogoro, Tanzania

**Keywords:** *T. solium* cysticercosis, Hair cortisol, Dehydroepiandrosterone (DHEA)

## Abstract

The aim of this study was to measure hair cortisol and dehydroepiandrosterone (DHEA) concentrations in naturally *Taenia solium* infected and non-infected control pigs and assess the effect of an environmental change on the aforementioned parameters.

Three hair patches were obtained from 13 *T. solium* infected and 15 non-infected controls sows, respectively corresponding to 3 time points (prior to, at and approximately two weeks after arrival at the research facility). Cortisol and DHEA were extracted using methanol and analysed by radio immune assay.

Mean hair cortisol concentrations were significantly lower (p < 0.001) in *T. solium* infected (4.7 ± 3.0 pg/mg) compared to control pigs (9.0 ± 3.7 pg/mg) prior to arrival at the research facility, however no significant difference was observed between the two groups at arrival and after approximately two weeks. Similar patterns were also observed for DHEA concentrations (infected pigs 253.9 ± 82.3 pg/mg, control pigs 387.7 ± 116.4 pg/mg) (p < 0.001).

Results showed that lean animals had significantly higher cortisol concentrations in both groups, infected and controls pigs, while DHEA was not significantly different between lean and normal animals.

Results of this study have shown that an environmental change could have an effect on pigs’ hormonal levels suggesting an undergoing adaptation process. After the pigs were kept under the same conditions, fed and watered *ad libitum*, no significant differences were observed between the groups, but a drop in DHEA concentrations was observed in all the pigs. Weight however had an effect on cortisol levels as lean animals had significantly higher cortisol concentrations in both groups, compared to normal pigs.

## Highlights

• Hair is a reliable matrix for measuring cortisol and DHEA concentrations in pigs.
• Both steroids were lower in infected than control pigs only in segment 1.
• Lean animals had significantly higher cortisol in both infected and control pigs.

## Graphical abstract

**Figure:**
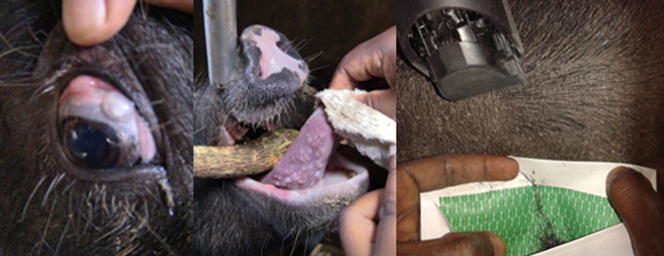

## 1 Introduction

Cortisol and dehydroepiandrosterone (DHEA) are steroid hormones, both synthesized from pregnenelone, the master steroid hormone, which is derived from cholesterol (Fallahsharoudi et al., 2015, Payne and Hales, 2004). Cortisol is a glucocorticoid produced by the adrenal cortex in response to adrenocorticotropic hormone (ACTH) secretion. Its actions are numerous and include stress response, increase blood sugar through gluconeogenesis, suppression of the immune system, and aids in the metabolism of fat, protein and carbohydrates (Marieb and Hoehn, 2010). DHEA is the most abundant circulating steroid hormone and is produced in the adrenal glands, the gonads and the brain (Ganong, 2005). It is a precursor for the synthesis of anabolic and sexual steroids (Kroboth et al., 1999). Studies suggest that variations in DHEA levels are associated with chronic health disorders (Abbasi et al., 1998, Tagliaferro and Ronan, 2001). Depletion of this hormone has been associated in humans with development of chronic unhealthy conditions (Tagliaferro and Ronan, 2001), moreover, experimental evidence strongly suggests that DHEA is closely linked to health maintenance (Tagliaferro and Ronan, 2001). The cortisol to DHEA ratio modulates biological energy output, and its effects are felt at the cellular level all over the body (Ganong, 2005). DHEA and cortisol are both hormones related to resilience and allostatic load (Charney, 2004). A variety of biological matrixes (plasma, saliva, faeces, urine, milk and hair) have widely been used for measuring cortisol and DHEA concentrations in a number of species (Cook, 2012, Russell et al., 2012). Serum and saliva samples provide a measurement of steroids concentration at a single point in time; hence both matrices are useful to test acute changes. However the act of sample collection can often interfere with the results, as it can be stressful by itself (Davenport et al., 2006).

Hair analysis has increasingly been used as a non-invasive method to obtain information on hypothalamic-pituitary adrenal axis (HPA-axis) activity in medium and long term periods and to assess adaptation to potentially stressful environmental changes (Bacci et al., 2014, Battini et al., 2015, Davenport et al., 2006, Macbeth et al., 2010, Peric et al., 2013, Peric et al., 2016, Stradaioli et al., 2017). Furthermore, steroids measurement in hair allows a long-term evaluation of chronic stress with a negligible influence of acute stress events (Novak et al., 2013). Hair sampling is a non-invasive, easy and painless procedure and has a low level of invasiveness associated with the sample collection. Hair samples are easy to store (stable for years at room temperature) and transport (Russell et al., 2012).

Several authors have reported the correlation between altered physiologic status and high hair cortisol concentrations (Burnett et al., 2015, Comin et al., 2012a, Comin et al., 2012b, Comin et al., 2013, Corradini et al., 2013). Galuppi et al. (2013) studied the relation between *Microsporum canis* infection in cats and hair cortisol concentrations, but so far no one has considered parasitic loads and hair steroids concentrations (Galuppi et al., 2013). The DHEA assay was validated in hair samples (Chen et al., 2013, Gao et al., 2013) however, to our knowledge, no study has been performed on hair DHEA concentrations in pigs.

It is known that parasites can alter steroids production. In fact, it was demonstrated that adrenal hormones exert a profound effect on several parasites determining the susceptibility, the course and severity of parasite infections (Escobedo et al., 2004, Romano et al., 2015). In most cases the infection disturbs the host environment, activates immune responses that end up affecting the endocrine system (Valdez et al., 2006, Valdez et al., 2014).

*T. solium* is a zoonotic parasite with the pig as intermediate host. Pigs ingest parasite eggs and develop cysticerci throughout muscles, heart and brain. When these reach the central nervous system, the condition is called neurocysticercosis (Garcia et al., 2003). Only few studies have shown the effect of *T. solium* infection on hormonal levels. Cardenas et al. (2012) observed lower plasma DHEA concentrations in human infected with *T. solium* compared with controls, whilst Pena et al. (2007) studied naturally *T. solium* infected boars but found no significant differences in cortisol and DHEA plasma concentrations.

As the parasite load is seen as chronic condition, hair matrix was used to study its effect on cortisol and DHEA concentrations.

The aim of this study was to evaluate the allostatic load in naturally *T. solium* infected and control pigs by measuring hair cortisol and DHEA concentrations and assess the effect of parasitoses and an environmental change on hair steroids concentrations.

## 2 Materials and methods

### 2.1 Ethics

Practices employed in the study were approved by Sokoine University of Agriculture (SUA), Morogoro, Tanzania (Ref. No. RPGS/R/AS/42/2014). All necessary steps to minimize animal suffering during transportation, housing and handling were applied in accordance with the national guidelines of ethics for health research and to the Animal Welfare Act (2008) (Mashalla et al., 2009, The United Republic of Tanzania, 2008).

### 2.2 Animals and study design

The study was performed at SUA, Morogoro, Tanzania. The study population consisted of 13 *T. solium* infected and 15 non-infected control sows with mean age (±standard deviation, SD) of 14 ± 7 months, crossbreeds between local and large white. Naturally infected pigs were purchased in villages of Kongwa district, Dodoma region, Tanzania, an area where the disease is known to be highly prevalent, as pigs are free roaming and left to scavenge (Phiri et al., 2003). Infection was diagnosed by tongue examination (Dorny et al., 2004). Pigs with more than three cysts under the tongue were considered to be infected. As the sensitivity of the diagnostic method is low, non-infected pigs were purchased in villages of Morogoro rural district, Morogoro region, Tanzania where the prevalence of porcine cysticercosis is known to be low as pigs are usually kept confined and fed with commercial feed and kitchen leftovers (Makundi, 2012). At the end of the study, all animals were sacrificed and inspected for *T. solium* cysticerci at Department of Veterinary Pathology, Faculty of Veterinary Medicine, SUA to avoid false negatives and vice versa.

Pigs not sexually mature (younger than 6 months) and size (lower than 50 cm in height) or in poor body condition (under two thirds of the average weight of 40 kg for a healthy adult pig and with body condition score (BCS) less than 2.5) and visibly ill (covered with ectoparasites and/or with injuries) were excluded from the study.

The animals were housed at the experimental unit for a total of 40 days under equal conditions in pens (4 × 3.5 m) with cemented floor and walls. Four groups were formed based on the size of the animals mixing infected and control pigs. In this way were identified 12 normal (6 infected and 6 controls) and 16 lean pigs (7 infected and 9 controls). Mean weights are shown in Table 1.

Pens were daily cleaned. Animals were fed with commercial dry pig feed twice a day and water was provided *ad libitum*. Either of the forages *Leucaena leucocephala*, *Amaranthus spinosus* and *A. viridis* were provided every day. The mean room temperature of the stable was 25 °C. On arrival, pigs were treated with a subcutaneous injection of 0.3 mg/kg of ivermectin (ivermectin ALFAMEC ® 1% Batch No. 1305136-01) to eliminate possible confounders (hard ticks, lice, fleas, mites and worms). Treatment was repeated after 14 days (Barragry, 1987).

### 2.3 Sampling procedure

A patch of hair (20 × 30 cm) was collected from the lower back region of each pig. Electronic clippers were used to shave the hair close to the skin. In this study, measurements revealed an average growth of 1.6 ± 0.2 cm/month (growth speed >0.5 mm/day).

In total three patches of hair were obtained from each pig. The first and the second segment were made 14 days after the animals’ arrival at the research facilities, the third at 15 days after from the first two samples.

**Fig. 1:**
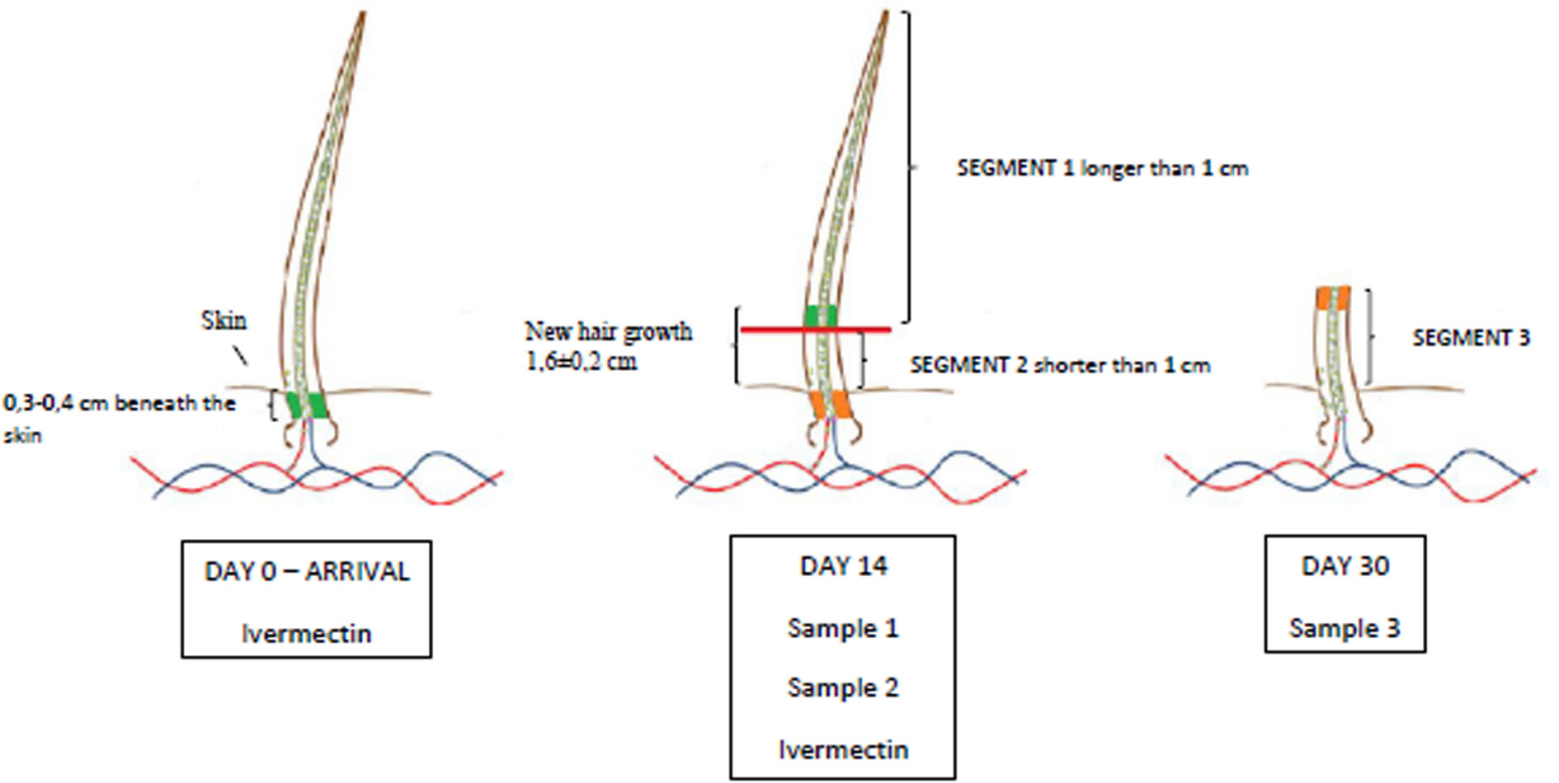
Sampling protocol (Russell et al., 2012). The coloured zones in the hair shaft (green and orange) show the speed growth of the hair. Sample 1, made at 14 days shaving off the hair at 1 cm from the skin, refers to hormonal productions prior to arrival at the research facility. Sample 2, made at 14 days shaving off the remaining 1 cm of hair, refers to the period immediately after the animals’ arrival, being made exclusively by the new hair growth. Sample 3, collected at day 30, corresponding to approximately two weeks after the pigs arrived at the research facility.

The first segment (longer than 1 cm) was obtained by shaving off the hair at 1 cm from the skin, following the collection procedure described by Kirschbaum et al. (2009). Considering the time of sampling and the growth speed, this first patch corresponded to the period prior to arrival at the research facility. The second segment (shorter than 1 cm) was obtained the same day by shaving off the remaining 1 cm of hair and corresponded to the arrival of the animals. In fact the section of hair located beneath the skin of the animals is never collected as hair is always shaved close to the skin and not plucked. The same approach was used in humans by Russell et al. (2012) and in horses by Montillo et al. (2014). Therefore, the hair steroids measurement in correspondence of the second segment referred to the period immediately after the animals’ arrival, being made exclusively on new hair growth. The third segment, obtained 30 days after the animals’ arrival, was made by shaving regrown hair. This sample corresponded to the period approximately two weeks after the animals’ arrival (Fig. 1).

Hair samples were kept in paper envelopes and stored at room temperature until analysis.

### 2.4 Hair extraction

Hair strands were washed in 5 ml isopropanol, as suggested by Davenport et al. (2006), and hair cortisol extracted according to the method described by Koren et al. (2002) with some modifications (Comin et al., 2012b). Approximately 60 mg of trimmed hair was placed in a glass vial along with 3 ml of methanol. The vials were incubated at 37 °C for 18 h. Next, the liquid in the vial was evaporated to dryness at 37 °C under an airstream suction hood. The remaining residue was dissolved in 0.6 ml of phosphate-buffered saline (PBS) 0.05 M, pH 7.5.

### 2.5 Hair analysis

Hair cortisol and DHEA concentrations were measured using a solid-phase microtitre RIA assay (Peric et al., 2013, Montillo et al., 2014). The cortisol intra- and inter-assay coefficients of variation (CV) were 3.8 and 9.4%, respectively. The cortisol sensitivity of the assay, calculated as the interpolated dose of the response to a concentration of zero minus the statistical error, was 1.23 pg/well.

The DHEA intra- and inter-assay CV were 4.6 and 10.9%, respectively. The DHEA sensitivity of the assay, calculated as the interpolated dose of the response to a concentration of zero minus the statistical error, was 0.62 pg/well.

The relations between the hair cortisol and DHEA and the respective standard curves determined through linear regression were linear, with a correlation coefficient of r = 0.99. The models were described by the equations y = 1.1470x + 0.9433 and y = 0.9123x + 4.54 for cortisol and DHEA, respectively.

### 2.6 Data analysis

Statistical analysis was performed using the R software environment for statistical computing and graphics (version 3.2.3). Cortisol and DHEA data were summarized by their arithmetic mean ± standard deviation (SD). Linear mixed-effects models were used to evaluate the relationship between changes in cortisol and DHEA in relation to presence of infection (infected or non-infected), sampling point (prior to, at and 30 days after arrival at the research facility) and pig status (lean or normal). The animal identification number (id) was included as random effect variable. A multivariate final model was obtained through a backwards selection procedure, at a significance level for removal of 5% (Pinheiro et al., 2016). A Tukey analysis was carried out as post hoc test.

## 3 Results

The linear mixed-effects models revealed significant effects for presence of infection, sampling point and pig body condition.

**Fig. 2:**
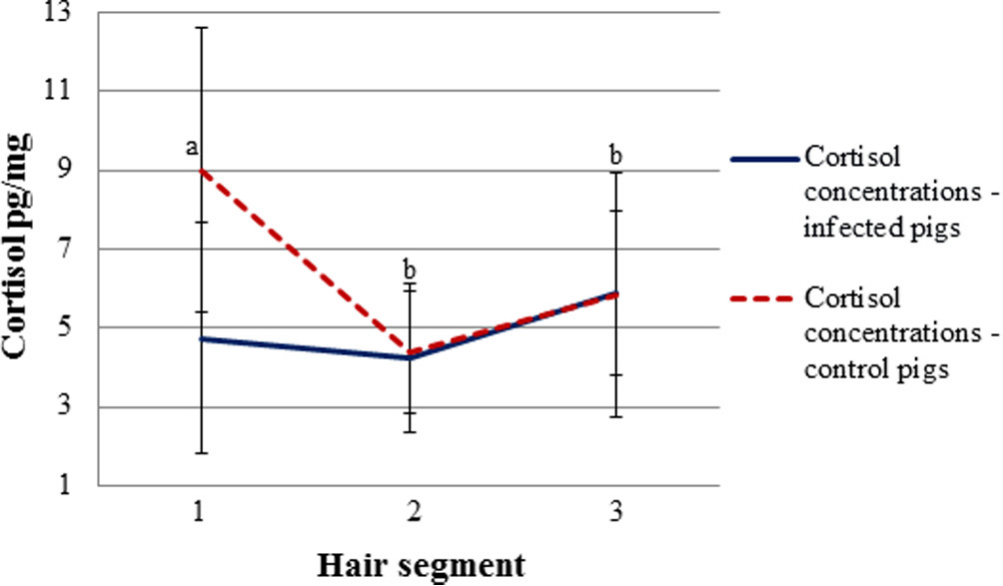
Mean ± standard deviation (SD) of hair cortisol concentrations in *T. solium* infected and control pigs at three different sampling points. (Unlike letters show significant difference between experimental groups (p < 0.05)).

Mean cortisol concentrations were significantly lower in *T. solium* infected (mean 4.7 ± SD 3.0 pg/mg) compared to control pigs (9.0 ± 3.7 pg/mg) (p < 0.001) in correspondence of the first segment, however no significant difference was observed between the two groups in the second and third segment taken on day 14 and 30 after arrival (Fig. 2). Cortisol concentrations dropped significantly (p < 0.001) in the control pigs from the first to the second segment. A significant difference was observed between lean and normal pigs (Table 1), with the lean pigs having significantly higher cortisol concentrations (p < 0.001) compared to the group of normal pigs at all three sampling points (Table 2).

**Fig. 3:**
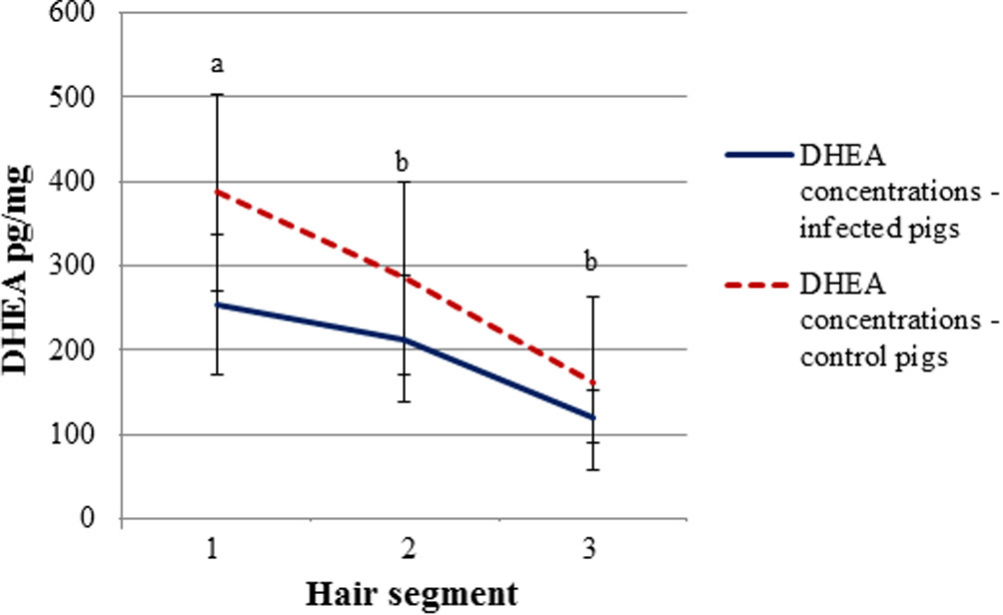
Mean ± standard deviation (SD) of hair DHEA concentrations in *T. solium* infected and control pigs at three different sampling points. (Unlike letters show significant difference between experimental groups (p < 0.05)).

Mean DHEA concentrations were significantly lower in *T. solium* infected (253.9 ± 82.3 pg/mg) compared to control pigs (387.7 ± 116.4 pg/mg) (p < 0.001) in correspondence of the first segment, however no significant difference was observed between the two groups in the second and third segment (Fig. 3). DHEA concentrations dropped significantly (p < 0.001) in all the pigs over time. No difference in DHEA concentrations were observed between lean and normal pigs.

The cortisol over DHEA ratio (Table 3) showed significant difference in both, infected and control pigs from the first and the second segment to the third (p < 0.001) and between lean and normal pigs (Table 4). However, no significant difference was observed between infected and control pigs.

## 4 Discussion

This is the first study that has assessed concentrations of both cortisol and DHEA using hair in a population of pigs naturally infected with *T. solium* and matching controls.

Results of this study have shown that after the pigs were kept under the same conditions, fed and watered *ad libitum*, no significant differences were observed between infected and control pigs. A drop of DHEA concentrations was observed in all the animals and weight showed to have an effect on cortisol levels as lean animals had significantly higher cortisol concentrations in both groups (p < 0.001), compared to normal pigs.

*Taenia solium* cysts can lodge in all parts of the pig’s body, including the central nervous system. A number of researchers have reported cysts to be responsible for immuno inflammatory changes that might alter the hormonal status of an animal, reducing testosterone and 17-β-estradiol or DHEA concentrations (Cardenas et al., 2012). This is in line with what was observed in this study, where DHEA hair concentrations in correspondence of segment 1 where higher in controls compared to infected pigs (p < 0.001). Pain, inflammation and other severe symptoms occurring in neurological diseases can result in chronic stress that can further reduce the function of several systems (Cardenas et al., 2012). The latter was observed in results of this study where at the moment of arrival pigs with infection had lower concentrations of cortisol in hair compared with healthy controls. These results might be explained by a reduced function of the HPA-axis in infected pigs, a pattern that has been observed in other studies where study subjects had an infectious or neurological disease (Cardenas et al., 2012). Differences in cortisol and DHEA concentrations between experimental groups occurred only prior to arrival at the research facility, while no significant differences were observed between the two groups in correspondance with segment 2 and 3. This might be explained by the fact that after arrival at the research facility all animals were treated with ivermectin to eliminate possible confounders (hard ticks, lice, fleas, mites and nematodes) and were kept under the same conditions. The animals were fed twice a day, water was provided *ad libitum* and foraging material was made available. This environmental change might have positively affected the pigs, hence the observed drop in cortisol concentrations and improved wellbeing on the pigs. The effect of an environmental change on hormone levels was also observed in a study on heifers, where the transfer from the valley farm to summer pastures had a significant effect of cortisol levels of the animals. After acclimatization, low cortisol levels were detected suggesting minimal involvement of the HPA-axis along with the wellbeing of the animals (Comin et al., 2011).

In a study on boars naturally infected with *T. solium*, Pena et al. (2007) found a reduction of sex hormones estradiol and testosterone, but found no significant differences in cortisol and DHEA concentrations. The latter might be explained by the fact that steroid hormones were measured in serum (Pena et al., 2007), while in our study, cortisol and DHEA concentrations were measured in hair. Hair in fact is not influenced by acute effects (Meyer and Novak, 2012), as happens for saliva or serum samples. In this study hair was also deemed more suitable as hair samples were easy to store and did not need to be refrigerated, which can be a challenge in low-income countries.

When looking at DHEA concentrations, these were significantly lower only in correspondance with segment 1 between infected and control pigs, while no significant differences were observed in correspondance with segment 2 and 3. In a study on human neurocysticercosis, infected humans showed significantly lower DHEA concentrations compared to healthy controls (Cardenas et al., 2012). Other studies also showed an impairment of the adrenal function as a result of an infectious disease or other diseases that caused severe illness (Hucklebridge et al., 2005, Leal et al., 2003, Parker et al., 1985), however to our knowledge studies where the effect of severe illness was monitored over time have not been performed. Only Peric et al. (2015) observed dairy cows over a period of 2 months and revealed an increase in hair DHEA concentrations during grazing compared to the time when the animals were kept indoors, while in our study we observed a significant drop of DHEA concentrations in all the pigs over time. Considering that experimental evidence strongly suggests that DHEA is closely linked to the maintenance of health (Tagliaferro and Ronan, 2001) and depletion of this hormone has been associated in humans with development of chronic unhealthy conditions (Tagliaferro and Ronan, 2001), we are witnessing a worsening of the pigs condition in terms of DHEA concentrations. The latter could be explained by the numerous environmental changes such as the arrival to the research facility, the formation of groups and new feeding.

Another important element observed in this study was the animals’ weight. Results showed that lean pigs had significantly higher cortisol concentrations in both groups, infected and controls pigs, while DHEA was not significantly different between lean and normal animals. Cooper et al. (2009) found low weaning weight was associated with lower corticosteroid-binding globulin concentrations and higher free cortisol in pigs, while Hillmann et al. (2008) observed that with increasing body weight average cortisol concentration increased and the circadian pattern became more pronounced in pigs (Cooper et al., 2009, Hillmann et al., 2008).

The cortisol over DHEA ratio showed significant difference in both, infected and control pigs from segment 1 and 2 to segment 3. An increased ratio suggested that an elevated metabolic effort was needed to cope with the new environment, in terms of housing, feeding and regrouping. These results disagree to those obtained by Peric et al. (2015) where dairy cows maintained the resilience although the animals underwent an important change of environment being moved from indoor winter housing to outdoor summer grazing on pastures (Peric et al., 2015). Our results also showed lean animals to have a lower level of resilience. This might be explained by the fact that lean animals had higher cortisol levels compared to normal pigs, while no difference was seen in DHEA concentrations, affecting hereby the cortisol to DHEA ratio. A similar change in ratio was also observed by Parker et al. (1985) in a study of severely ill human patients, where the urine and serum results demonstrated a relative shift of pregnenolone metabolism away from DHEA and towards cortisol.

## 5 Conclusion

Results of this study have shown that environmental changes, like housing, feeding and re-grouping, could have an effect on the hormonal levels of the pigs suggesting an undergoing adaptation process. After the pigs were kept under the same conditions, fed and watered *ad libitum*, no significant differences were observed between the groups in cortisol and DHEA hair concentrations. A drop in DHEA concentrations was observed in all the animals in the three segments. This could due to the effects of the numerous environmental changes such as the arrival to the research facility, the formation of groups and the new feeding.

Results showed that lean pigs had significantly higher cortisol concentrations in both groups, infected and controls pigs, while DHEA was not significantly different between lean and normal animals.

This is the first study that treated the interaction between parasitoses and chronic alteration of adrenal steroids and more studies are needed to assess the interaction.

## Author contributions

Conceived and designed the experiments: Trevisan C, Montillo M, Prandi A, Johansen MV. Collected the samples: Trevisan C.; Contributed reagents/materials/analysis tools: Montillo M, Prandi A.; Analysed the samples: Montillo M. Analysed the data and performed the statistical analysis: Trevisan C, Montillo M.; Wrote and revised the paper: Trevisan C, Montillo M, Mkupasi E, Ngowi H, Prandi A, Johansen MV.

## Figures and Tables

**Table 1:** Mean body weight (kg) ± standard deviation (SD) of *T. solium* infected and control pigs in the lean and normal group at four different measurement days.

Pen	Group	Day 0	Day 9	Day 25	Day 33
Normal	Infected	66.2 ± 11.0^a^	71.8 ± 13.0^a^	78.3 ± 17.2^a^	81.7 ± 19.5^a^
	Control	51.8 ± 8.1^a^	59.0 ± 9.0^a^	62.3 ± 9.2^a^	64.8 ± 10.1^a^

Lean	Infected	43.0 ± 6.6^b^	48.6 ± 9.2^b^	50.9 ± 9.5^b^	52.0 ± 4.5^b^
	Control	38.8 ± 2.6^b^	43.3 ± 2.9^b^	49.0 ± 5.2^b^	51.4 ± 11.3^b^

SD – standard deviation.

^a,b^: Factor with unlike letters differ significantly (p < 0.05).

**Table 2:** Mean cortisol concentrations (pg/mg) ± standard deviation (SD) of lean and normal pigs in three hair segments.

Pigs	Hair segment		
	1	2	3
Normal	5.0 ± 2.7^a^	3.3 ± 1.2^a^	4.1 ± 0.9^a^
Lean	8.5 ± 4.0^b^	5.1 ± 1.6^b^	7.1 ± 2.8^b^

^a,b^: Factor with unlike letters differ significantly (p < 0.05) within the same column.

**Table 3:** Cortisol/DHEA (^*^100) ratio ± standard deviation (SD) of *T. solium* infected and control pigs in three hair segments.

Pigs	Hair segment		
	1	2	3
Infected	2.1 ± 1.6^a^	2.5 ± 1.9^a^	5.3 ± 2.6^b^
Controls	2.5 ± 1.2^a^	1.7 ± 0.7^a^	4.5 ± 2.9^b^

^a,b^: Factor with unlike letters differ significantly (p < 0.05) within the same row.

**Table 4:** Cortisol/DHEA (^*^100) ratio ± standard deviation (SD) of lean and normal pigs in three hair segments.

Pigs	Hair segment		
	1	2	3
Normal	1.6 ± 0.8^a,*^	1.4 ± 0.8^a,*^	2.9 ± 1.2^a,+^
Lean	2.9 ± 1.6^b,*^	2.5 ± 1.7 ^b,*^	6.3 ± 2.8^b,+^

^a,b^: Factor with unlike letters differs significantly (p < 0.05) within the same column.

^+,*^: Factor with unlike symbol differs significantly (p < 0.05) within the same row.
